# Data-Driven Inverse Design Enables a Dexterous Hand with Human-Comparable Dynamic Performance in Structured Tasks

**DOI:** 10.3390/biomimetics11060434

**Published:** 2026-06-18

**Authors:** Lei Jiang, Kaixin Lan, Xianwei Liu, Chaojie Fu, Yongbin Jin, Hongtao Wang

**Affiliations:** 1 Center for X-Mechanics, Zhejiang University, Hangzhou 310012, China; 12524001@zju.edu.cn (L.J.); 22424122@zju.edu.cn (K.L.); 3140104175@zju.edu.cn (X.L.); 12124058@zju.edu.cn (C.F.); 2 State Key Laboratory of Fluid Power and Mechatronic System, Zhejiang University, Hangzhou 310012, China; 3 ZJU-Hangzhou Global Scientific and Technological Innovation Center, Zhejiang University, Hangzhou 311200, China

**Keywords:** dexterous hand, data-driven design, inverse design, biomimetic robotics, high-dynamic performance, tendon-driven actuation, anthropomorphic hand

## Abstract

The design of dexterous robotic hands has long been constrained by empirical paradigms that struggle to balance anthropomorphic fidelity with dynamic performance. This study aims to establish a systematic methodology that bridges this gap through data-driven inverse design. We construct a quantitative association map between design variables and performance metrics using a comprehensive dataset of existing dexterous hands, then apply this map to translate explicit high-frequency dynamic targets into an optimized hardware configuration. The analysis reveals that the dominant principles for high-speed performance—tendon-driven transmission, proximal actuation, and lightweight rigid structures—closely mirror the biomechanical architecture of the human hand. Guided by this convergence, we develop the Beyond Hand, a 20-degree-of-freedom (DoF) anthropomorphic hand that preserves human-scale dimensions. Standardized frequency-response tests across all 15 joints show magnitude attenuation below 3 dB at 14 Hz and cutoff frequencies clustered around 10 Hz. In rhythm-game and Tetris-style manipulation tasks, the hand maintains over 90% accuracy at actuation frequencies up to 12 Hz. These results demonstrate that a performance-driven pathway can systematically elevate the dynamic capabilities of humanoid dexterous hands, offering a scalable framework for biomimetic robotic design.

## 1. Introduction

The design of dexterous robotic hands has advanced considerably over the past two decades, yet a fundamental methodological challenge persists. The prevailing design paradigm remains predominantly empirical: engineers iteratively refine prototypes through intuition and trial-and-error, a process ill-suited to the high-dimensional, tightly constrained design space that dexterous hands occupy [1,2,3]. This approach inevitably embeds compromises among critical performance indicators, and as a result, existing dexterous hands tend to cluster into two distinct design philosophies. One category prioritizes anthropomorphic fidelity—emphasizing kinematic similarity and high DoF—but often at the expense of dynamic speed and responsiveness [4]. The other achieves impressive speed or force output through compact, simplified mechanisms, yet sacrifices human-like scale, dexterous versatility, and coordinated multi-finger control [5,6]. Bridging this divide—achieving a design that simultaneously maintains human-like size and dexterity while substantially elevating dynamic performance—has remained an open and difficult problem.

Recent advances in biomimetic structures, lightweight materials, actuation modalities, and transmission mechanisms have significantly broadened the accessible design space [7,8,9,10,11,12]. However, these innovations have largely been absorbed into the same forward-design framework: a specific configuration is proposed first, and its performance is evaluated afterward. What remains absent is a mature methodology capable of moving in the reverse direction—starting from explicit, quantitative dynamic performance targets and systematically deriving the optimal combination of actuation, transmission, and structural parameters. To address this gap, our group previously proposed a data-driven inverse design framework [13]. By aggregating design variables and performance metrics from a large corpus of existing dexterous hands, we constructed a “design variables—performance metrics” association map using Cramér’s V correlation analysis. This map provides quantitative, performance-targeted guidance for hardware design decisions. However, this framework was proposed only as a concept; it has never been validated on a complete humanoid dexterous hand system, and its potential to realize dynamic performance approaching or matching human-level performance on structured high-frequency tasks on physical hardware has remained untested.

The present study provides this critical validation. We apply the data-driven inverse design framework to develop, for the first time, a fully anthropomorphic dexterous hand optimized specifically for high-frequency dynamic performance. Starting from the quantitative association map, we identify an optimal region of the design space and translate it into a concrete hardware configuration. The resulting system, termed the Beyond Hand, retains a human-like 20-DoF architecture and anthropomorphic scale while achieving dynamic capabilities that, under the specific test conditions (rhythm-game and Tetris tasks), are comparable to or better than the best performance observed in our intermediate-level human participant cohort (*n* = 5) in terms of task completion speed and timing consistency. Its design embodies a biomimetics-informed engineering strategy: the key features independently identified through data-driven analysis—tendon-driven force transmission, proximal actuator placement, and lightweight skeletal structures—closely mirror the biomechanical organization of the human hand [14,15,16]. This convergence between statistical optimization and biological evolution suggests that data-driven inverse design can recover principles that nature itself has refined over millions of years, providing an independent line of evidence to support biomimetic design decisions.

The main contributions of this work are as follows:

1. First system-level validation of data-driven inverse design. We demonstrate that a quantitative design-performance association map can successfully guide the complete hardware development of a humanoid dexterous hand, translating explicit dynamic performance targets into an optimal physical configuration.
2. Development of the Beyond Hand platform. The resulting hand is a compact, 20-DoF anthropomorphic system that matches human scale while achieving enhanced high-frequency dynamic capability comparable to the best observed performance of our intermediate-level human participant cohort (*n* = 5) on the structured rhythm-game and Tetris tasks, with all actuators placed in the forearm to minimize distal inertia.
3. Rigorous experimental benchmarking against human baselines and existing designs. Through standardized frequency-response characterization across all 15 finger joints, rhythm-game tasks demanding millisecond-level multi-finger coordination at frequencies up to 12 Hz, and Tetris-style single-finger manipulation tests under a fixed wrist, we establish clear performance advantages in speed, timing accuracy, and high-frequency stability on the structured rhythm-game and Tetris tasks relative to our intermediate-level human participant cohort (*n* = 5) and established dexterous hand designs.

The remainder of this paper is organized as follows. Section 2 describes the materials and methods, including the data-driven inverse design framework, the biomimetics-informed derivation of the Beyond Hand configuration, and the experimental protocols for performance evaluation. Section 3 presents the results, covering the association map, the design outcome, standardized performance metrics, and high-dynamic manipulation tests. Section 4 discusses the implications of the convergence between data-driven and evolutionary design principles, the specific dimensions in which the Beyond Hand extends beyond biological capabilities, and the limitations and future directions of this work. Section 5 concludes the paper.

## 2. Materials and Methods

### 2.1. Data-Driven Inverse Design Framework

This work builds upon a dataset established in our prior study [13]. We have now expanded this dataset through a systematic search across Google, Google Scholar, ScienceDirect, IEEE Xplore, and Scopus, covering more than 370 articles. After screening for relevance, data completeness, and cross-validation across multiple sources, a total of 77 distinct dexterous hands were included in the final statistical analysis. The inclusion criteria required that each hand be either commercially available or described in sufficient detail in the literature, and that at least partial quantitative or semi-quantitative information could be extracted.

For data normalization, where different units or measurement methods were reported, we converted all values to consistent metric units (e.g., Newtons for force, degrees per second for speed, kilograms for weight, and dimensionless ratios for compactness). For missing and inconsistent data across sources, we applied a three-step strategy: first, reasonable completion based on similar designs or diagram measurements; second, exclusion of data points with excessive missing information or impossible speculation; third, merging of repeated information from different sources to ensure consistency. To minimize dataset bias, we included all identifiable dexterous hands that met the inclusion criteria, without any selective omission based on design preferences or performance outcomes.

The resulting dataset, comprising 77 distinct dexterous hands, systematically documents key design variables—including the number of DoF, driving form, transmission mode, structural form, and structural material—alongside five performance metrics: maximum speed, fingertip force, compactness, weight, and maximum single-finger motion range. The exact composition of the 77 hands and their respective data quality are shown in the table in Part 3 of the Supplementary Video S1.

To analyze the associations between design variables and performance metrics, we conducted a quantitative correlation analysis using Cramér’s V, a statistical measure appropriate for categorical data [13]. Prior to this analysis, all continuous performance metrics were converted into two discrete categories based on functionally meaningful thresholds derived from general data on human hand performance: fingertip force was classified as <12 N or ≥12 N; maximum speed as <200°/s or ≥200°/s; weight as <0.5 kg or ≥0.5 kg for hands without forearms, and as <1.5 kg or ≥1.5 kg for integrated hand-forearm systems; maximum single-finger motion range as below the human level or at/above it; and compactness (defined as the finger length-to-thickness ratio) as <5.5 or ≥5.5. These thresholds are grounded in typical human hand capabilities reported in the literature. We acknowledge that human hand performance varies considerably across individuals, and therefore the exact boundary between “low” and “high” categories is inherently somewhat uncertain. To evaluate the potential influence of threshold selection on the correlation results, we conducted a sensitivity analysis by varying each threshold within a reasonable range (e.g., ±10–20% of the original value) and recalculating Cramér’s V. The results showed that the strength and direction of the correlations remained consistent. Thus, the main conclusions of our correlation analysis are not sensitive to the exact choice of cutoff values.

Correspondingly, the key design variables were categorized as follows: the number of DoF was classified as <3 or ≥3; structural form was classified by motor location as built-in or external; driving form was classified as fully actuated or underactuated; and structural material was classified as metallic or non-metallic. Transmission modes were classified into first-level and second-level categories. The first-level mode refers to the transmission from the actuator to the finger, encompassing tendon-driven, gear-driven, ball screw-driven, and linkage-driven systems. The second-level mode refers to the transmission within the finger itself, which includes tendon-driven, gear-driven, belt-driven, and linkage-driven systems. For clarity in the subsequent analysis and visualization, transmission modes across both levels were consolidated into two representative categories: tendon-driven and linkage-driven.

The resulting correlation matrix was visualized as a Sankey diagram, which maps the couplings between design variables and performance metrics. The width of each flow corresponds to the calculated Cramér’s V value, with wider links indicating stronger correlations. Full results of this analysis are presented in Section 3.1.

### 2.2. Biomimetics-Informed Inverse Design

We take a high-dynamic rhythm-based game task as the specific design target. This task imposes multiple stringent requirements on the dexterous hand, including extreme speed, high acceleration, structural compactness, and wide response bandwidth—all of which correspond directly to the performance metrics analyzed in the association map.

The key design principles emerging from the data-driven analysis—the strong correlation between tendon-driven transmission and speed, the benefits of lightweight metallic structures for force output, and the trade-off between DoF count and system weight—point unequivocally toward a specific region of the design space. To understand why this particular combination of features emerges as optimal, we examine the anatomical basis of the human hand’s remarkable dynamic capabilities. The human hand achieves its combination of speed and dexterity primarily through a tendon-driven architecture: long flexor and extensor tendons, originating from muscle bellies located in the forearm, transmit forces across multiple joints to the phalanges. This arrangement confers two critical advantages. First, by placing the majority of muscle mass proximally in the forearm rather than within the hand itself, the mass and inertia of the distal segments are minimized, enabling rapid acceleration and direction changes. Second, the multi-articular routing of tendons across successive finger joints provides a natural mechanical coupling that simplifies coordinated motion patterns while maintaining compliant, energy-efficient force transmission. The convergence between these well-established biomechanical principles and the statistical patterns independently identified in our association map is striking: the data-driven analysis recovers, through purely quantitative means, the same design strategy that biological evolution arrived at over millions of years.

Guided by this dual foundation of biological insight and quantitative data, we derive the following configuration for the Beyond Hand. To achieve extreme speed, we select a tendon-driven mode as the first-level transmission, which closely mimics the long flexor tendons of the human hand and aligns with the map’s strong correlation between tendon drive and high-speed performance. To reduce transmission losses and increase structural stiffness, we adopt a linkage-driven mode as the second-level transmission. This hybrid tendon–linkage architecture parallels the human hand’s combination of flexible tendons for force transmission and rigid skeletal segments for structural support. To balance fingertip force and structural compactness, we use aluminum alloy as the main structural material, analogous to the role of bone tissue in providing lightweight, high-strength frameworks. In terms of overall configuration, we maintain a human-like 20-DoF design and—following the biological strategy of reducing distal inertia—place all actuators in the forearm, remote from the hand itself. Table 1 summarizes the systematic mapping between these design choices and their biological counterparts [17,18].

### 2.3. Standardized Performance Evaluation Methods

To characterize the frequency response and key physical capabilities of the Beyond Hand, a series of standardized benchtop tests were conducted.

#### 2.3.1. Frequency Response Characterization

Sinusoidal frequency-sweep experiments from 1 to 16 Hz were performed on all joints of the five fingers. The test encompassed the metacarpophalangeal (MCP), proximal interphalangeal (PIP), and abduction/adduction (ABAD) joints of each finger; for the thumb, the corresponding carpometacarpal (CMC), MCP, and interphalangeal (IP) joints were evaluated. Motion amplitudes were set to ±20° for abduction/adduction joints and 0–80° for flexion/extension joints, consistent across all fingers. Command trajectories were sent at 100 Hz, and joint angle sensors sampled at 160 Hz. The cutoff frequency for each joint was identified using the standard criteria of a −3 dB magnitude drop and a −90° phase lag. Each frequency sweep test was repeated three times per joint. The three runs produced highly consistent results; the best-performing run (i.e., with the most stable tracking and lowest phase lag) is presented as representative.

Each joint of each finger is driven by an HT2806 motor (rated torque 0.04 N·m, rated speed 1200 RPM, maximum no-load speed 2200 RPM, stall torque 0.14 N·m; Changzhou Hetai Motor & Electrical Appliance Co., Ltd., Changzhou, China). The tendon is wound around a 5 mm diameter shaft connected to the motor. A cascaded PID control structure is implemented on an STM32F407 microcontroller (STMicroelectronics, Geneva, Switzerland). The inner current loop runs at 20 kHz, the middle velocity loop at 20 kHz, and the outermost position/impedance loop at 1000 Hz. During the 1–16 Hz sweep tests, the system operated in position-controlled mode with a sinusoidal reference trajectory.

The position control loop updates at 1000 Hz. The round-trip communication latency between the host computer and the lower-level controller was measured as 3.6 ± 0.3 ms. The total system pure delay from command issuance by the host computer to joint motion feedback via the encoder was measured as 12 ± 4 ms. A proportional controller tuned for critical damping (Kp = 60) was used for the outermost position loop; the inner velocity and current loops employ PI control. No electronic damping was applied; the system relies primarily on the natural mechanical damping of the joints.

#### 2.3.2. Key Performance Metrics and Benchmarking

In addition to frequency response, the peak angular velocity of the PIP joint, maximum fingertip force under a bent posture, system weight, finger compactness ratio (finger length-to-thickness), and fingertip repeatability were measured. To contextualize these results, a systematic benchmarking was performed against both the human hand and three representative state-of-the-art dexterous hands (detailed in Section 3.3).

#### 2.3.3. Radar-Chart Normalization Method

For the radar-chart comparison in Section 3.3.3, all six performance indicators (DoF ratio, fingertip force ratio, weight ratio, speed ratio, compactness ratio, and frequency response ratio) were normalized to the human hand as the baseline, where the human hand is assigned a value of 1. Specifically, each ratio was computed as: Ratio = (value of the dexterous hand)/(corresponding value of the human hand). No additional weighting was applied; all six metrics were treated equally to provide an unbiased, unweighted comparison across designs.

The benchmark dexterous hands (Inspired Hand, Shadow Hand, DLR-HIT II Hand) were tested under different experimental conditions and reported in separate literature sources. Therefore, direct comparison under identical testing protocols is not feasible. The radar chart is intended as an indicative, qualitative comparison based on the best available data reported in the literature, rather than a strictly controlled benchmarking study.

### 2.4. High-Dynamic Manipulation Task Design

To assess the Beyond Hand’s performance under realistic manipulation scenarios, two experimental tasks were designed, each targeting a distinct aspect of dynamic capability.

#### 2.4.1. Rhythm-Game Task

A music rhythm-game task was employed to evaluate multi-finger coordination and high-speed dynamic response. The robotic hand was required to execute 15 different tapping patterns according to sliding-bar cues on a screen, pressing the corresponding key within a strictly constrained time window upon slider arrival at a judgment area. The time deviation between each press and the target beat was categorized into four levels: ±50 ms (“Best”), ±50–100 ms (“Good”), ±100–150 ms (“Cool”), and >150 ms (“Miss”). Overall accuracy was defined as the proportion of presses rated “Best” when all presses were ideally timed. The total slider density reached up to 34 notes per second, with a maximum single-lane density of 12 notes per second. The robotic hand performed the task three independent times. The outcomes across the three runs were similar; the best result (the run with the highest overall accuracy) is reported. A video demonstration is provided in the Supplementary Materials (Video S1, part 1).

#### 2.4.2. Tetris-Style Manipulation Task

A single-finger dynamic experiment was designed based on a Tetris game to evaluate high-speed single-finger agility and reachable workspace under a fixed wrist. The middle finger alone performed rapid presses on the “↑” key (first row) and “←” and “→” keys (second row). The block descent speed increased progressively: 150 ms per grid cell (0–30 s), 120 ms (31–45 s), 100 ms (46–60 s), and 75 ms (61–75 s). The task was repeated three times under the same conditions. The results were consistent across repetitions; the best performance (the run with the fastest and most accurate key presses) is reported. A video demonstration is provided in the Supplementary Materials (Video S1, part 2).

### 2.5. Human Participant Baseline

To benchmark the Beyond Hand against human performance, five healthy doctoral students (age range 24–27 years, mean 25.2 years) participated in the rhythm-game task. Their skill level with rhythm games was intermediate: they could achieve high scores on pieces of moderate difficulty but performed considerably worse on fast-tempo tracks.

Each participant performed 10 repeated trials of the same manipulation tasks. For each participant, the best outcome across the 10 trials was recorded as the representative human performance, as our aim was to compare the upper bound of human performance with that of the robotic system under identical task conditions. Therefore, we report the individual best performance rather than a group mean with variance.

Written informed consent was obtained from all participants prior to the experiments. The study was conducted in accordance with the ethical guidelines of our institution. Under our institutional policies, this low-risk behavioral study (non-invasive, healthy adult volunteers) was exempt from formal ethics committee review.

## 3. Results

### 3.1. Association Map Between Design Variables and Performance

**Figure 1:**
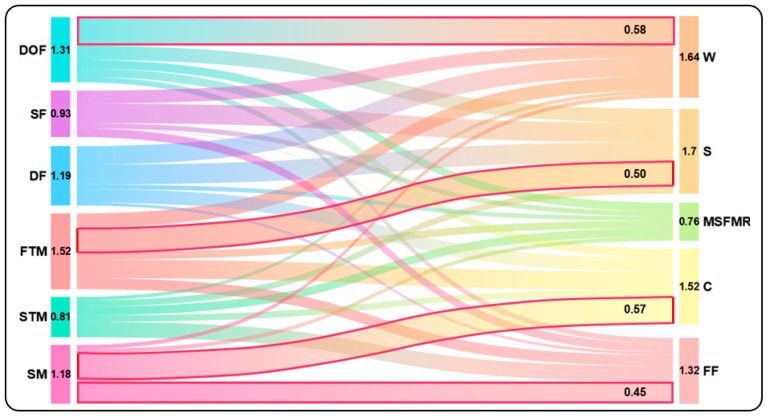
The Sankey diagram of the correlation value between the design variables and the performance of the dexterous hand. FTM: First-level Transmission Mode; STM: Second-level Transmission Mode; DF: Driving Form; SF: Structural Form; SM: Structural material; W: Weight; S: Speed; C: Compactness; MSFMR: Maximum single finger motion range; FF: Fingertip force. The four red boxes indicate the four pathways with the highest correlation.

The resulting Sankey diagram (Figure 1) visualizes the quantitative couplings between design variables and performance metrics. Each flow width reflects the magnitude of the Cramér’s V correlation coefficient; values between 0.40 and 0.70 indicate moderate-to-strong statistically relevant relationships [19].

Several strong associations emerged from the analysis. The first-level transmission mode exhibited a pronounced impact on speed, with tendon-driven configurations proving particularly effective for high-speed motion. Metal materials, owing to their high stiffness, were associated with improvements in both output force and structural compactness. The number of DoF showed a clear positive correlation with system weight. The analysis also clarified a common misconception: blindly increasing the number of actuated DoF or transmission stages introduces extra mass and frictional losses, which degrade dynamic performance.

Notably, the dominant design principles surfacing from this purely data-driven analysis—tendon routing for speed, proximal mass concentration, and rigid lightweight structures—closely mirror the biomechanical architecture of the human hand. This convergence indicates that a tendon-driven first-level transmission, lightweight metallic structure, and distal-mass-minimizing actuator placement constitute the most promising combination for high-speed dynamic tasks. The following section describes how these principles were translated into a concrete hardware design.

### 3.2. Design Outcome: The Beyond Hand

**Figure 2:**
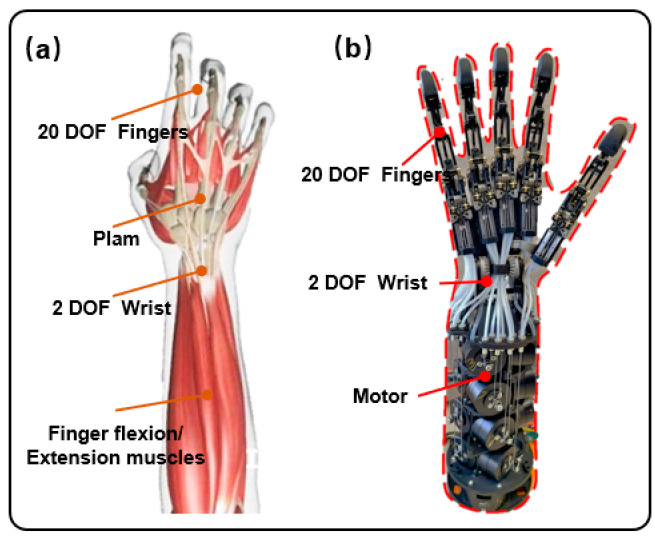
Comparison between the human hand and the Beyond Hand. (**a**) Distribution of finger and wrist DoF in the human hand; (**b**) Distribution of finger and wrist DoF in the Beyond Hand.

Following the inverse design process, we successfully developed the dexterous hand Beyond Hand (Figure 2). Consistent with the optimal configuration derived from the association map, the hand adopts a tendon-driven first-level transmission, linkage-driven second-level transmission, aluminum alloy structural components, and a 20-DoF anthropomorphic layout with all actuators placed in the forearm. This configuration embodies the key biomechanical principles identified in Section 2.2—proximal actuation for low distal inertia, tendon-driven force transmission for high-speed motion, and a lightweight rigid skeletal framework for structural efficiency.

The overall dimensions and kinematic structure closely match those of the human hand. Figure 2a illustrates the 21 DoF of the human hand (including the wrist), while Figure 2b shows the corresponding 20-DoF distribution in the Beyond Hand, with the thumb, index, middle, ring, and little fingers each equipped with multiple actuated joints, and the wrist providing two additional DoF for orientation adjustment. Table 1 (Section 2.2) summarizes the mapping between biological features and engineering choices.

### 3.3. Standardized Performance Evaluation

#### 3.3.1. Frequency Response

**Figure 3:**
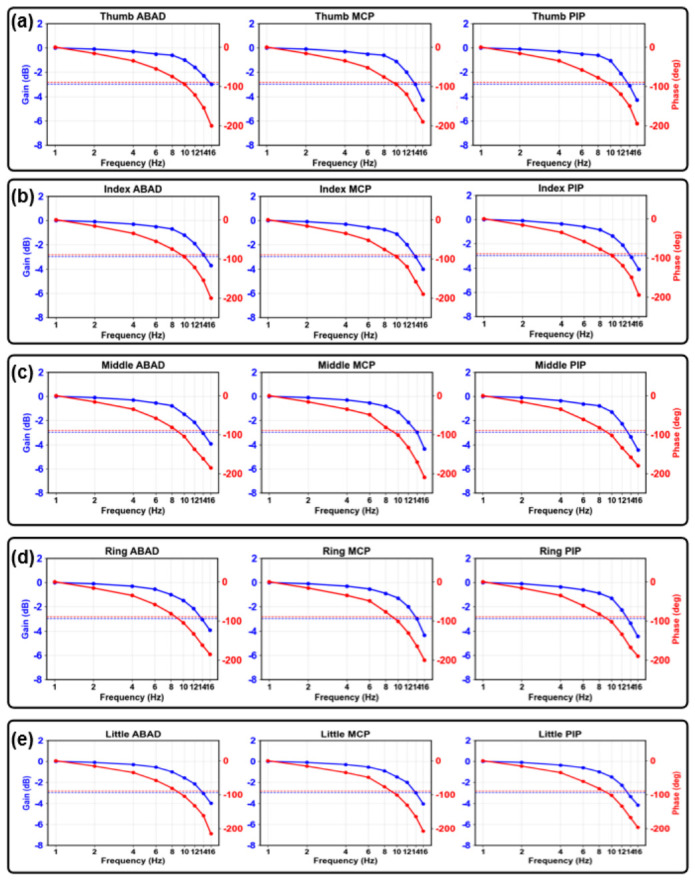
Bode plots of all finger joints under sinusoidal frequency-sweep excitation from 1 to 16 Hz: (**a**) thumb joints; (**b**) index finger joints; (**c**) middle finger joints; (**d**) ring finger joints; (**e**) little finger joints.

Bode plots were constructed for all 15 finger joints from the measured angle data (Figure 3). Using the standard criteria of a −3 dB magnitude drop and a −90° phase lag, the cutoff frequencies were found to be tightly clustered around 10 Hz, with all joints maintaining magnitude attenuation below 3 dB at 14 Hz. This uniformity across all five fingers indicates that the system possesses stable and well-balanced dynamic characteristics throughout the entire hand.

**Figure 4:**
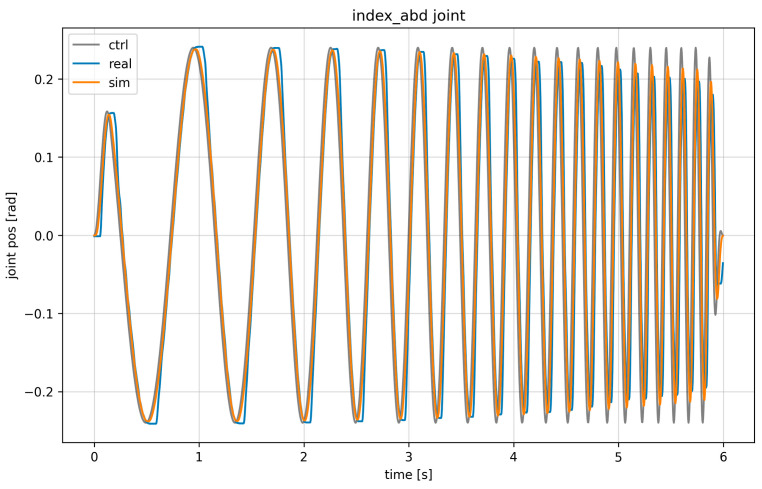
Dynamic hysteresis analysis of finger response from 1 Hz to 6 Hz. Lower frequencies show more noticeable hysteresis (phase lag and amplitude attenuation); higher frequencies exhibit reduced hysteresis effects.

To further examine the effect of tendon elasticity and friction on tracking accuracy, we performed a dynamic hysteresis analysis from 1 Hz to 6 Hz. As shown in Figure 4, at lower frequencies (1–3 Hz), hysteresis is more noticeable, leading to a small phase lag and amplitude attenuation. The controller remains stable and trajectory tracking is still satisfactory. At higher frequencies (5–6 Hz), hysteresis becomes less pronounced relative to the inertial and elastic effects.

#### 3.3.2. Measured Performance and Durability Test Results

**Figure 5:**
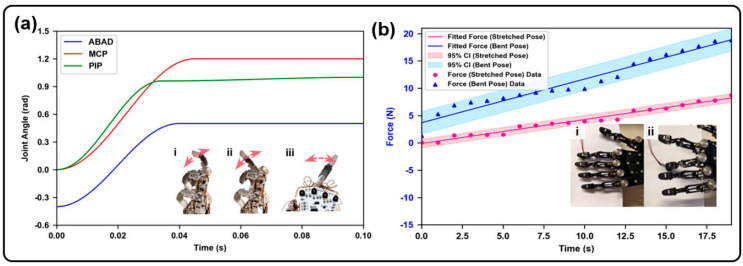
Measured performance metrics of the Beyond Hand. (**a**) The PIP joint achieved a peak angular velocity of 1910°/s. (**b**) A maximum fingertip force of 18 N was measured in a bent posture.

The absolute speed and force capabilities of individual joints established the system’s physical performance envelope. The PIP joint reached a peak angular velocity of 1910°/s (Figure 5a), and the maximum fingertip force under a bent posture was 18 N (Figure 5b). The hand weighs 2.1 kg, comparable to a human forearm-hand segment. The finger compactness ratio is 4.8.

**Figure 6:**
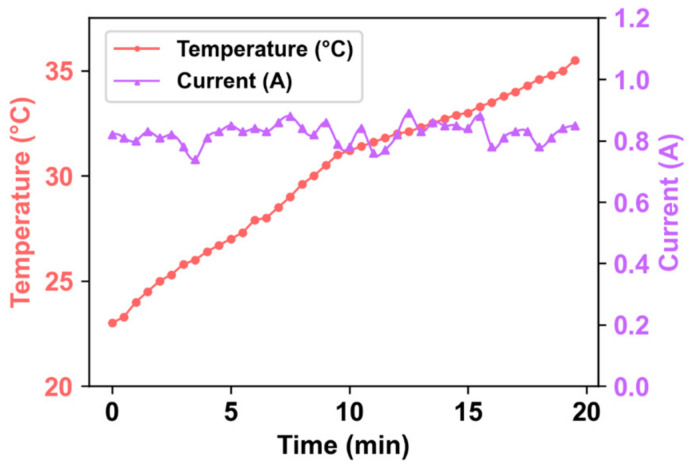
Motor temperature evolution during 20 min of continuous 6 Hz operation. Temperature rose from 23 °C to 36 °C, staying within the safe operating limit.

To evaluate long-term durability and thermal effects under sustained operation, we first ran the dexterous hand continuously at 6 Hz for 20 min (≈7200 cycles). Motor temperature rose from 23 °C to 36 °C, remaining well within the safe operating range (Figure 6). Tendon strings remained intact with no signs of breakage or significant elongation.

Next, we conducted a more extensive endurance test involving 30,000 repeated actuation cycles under continuous high-frequency key-pressing operation. After completing these 30,000 cycles, we measured the bidirectional repeatable positioning accuracy of a single finger joint (in terms of linear displacement at the fingertip) without any intermediate maintenance or part replacement. For the bending direction (flexion/extension), target joint angles were 15°, 30°, and 45°; for the lateral sway direction (abduction/adduction), target angles were 15° and 30°. For each target angle, the test was repeated ten times with fifteen data points per hold, yielding 150 data points per angle. Repeatability was defined as ±3σ.

After 30,000 cycles, the bending direction results were: at 15°, μ = 3.782 mm, σ = 0.0671 mm, repeatability = ±0.2013 mm; at 30°, μ = 5.821 mm, σ = 0.0695 mm, repeatability = ±0.2085 mm; at 45°, μ = 9.276 mm, σ = 0.0712 mm, repeatability = ±0.2136 mm. The overall repeatability for bending was ±0.2136 mm. For the lateral sway direction: at 15°, μ = 2.207 mm, σ = 0.0316 mm, repeatability = ±0.0948 mm; at 30°, μ = 3.632 mm, σ = 0.0341 mm, repeatability = ±0.1023 mm. The overall repeatability for lateral sway was ±0.1023 mm.

**Figure 7:**
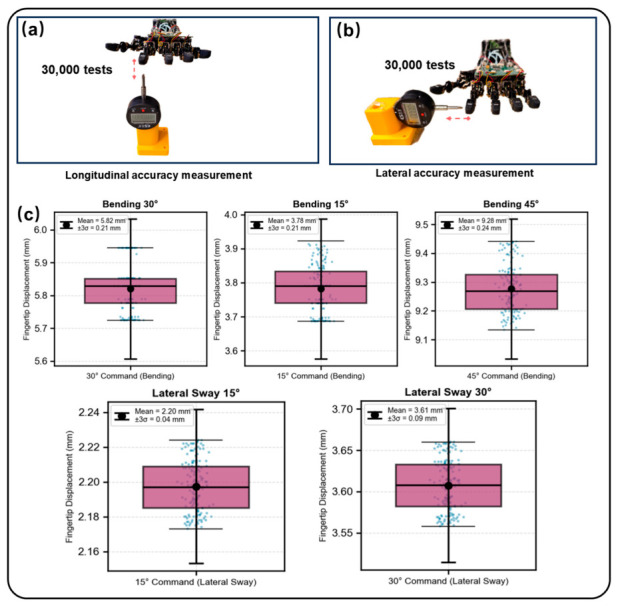
Repeatable positioning accuracy of a single finger joint after 30,000 cycles. (**a**) Bending direction repeatability (±3σ) at 15°, 30°, and 45° target angles. (**b**) Lateral sway direction repeatability (±3σ) at 15° and 30° target angles. (**c**) Fingertip position error distribution for all five test conditions.

Crucially, even after 30,000 cycles of continuous high frequency operation, the positioning accuracy remained consistently high (Figure 7). These results demonstrate that despite the use of tendon-driven transmission, the finger exhibits excellent long-term repeatability and durability.

#### 3.3.3. Radar-Chart Comparison

**Figure 8:**
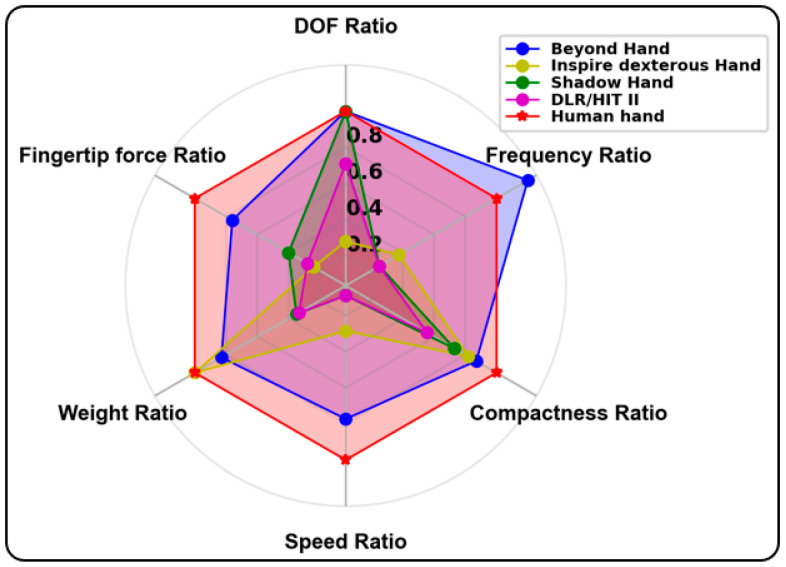
Radar-chart comparison across six normalized metrics for the human hand (human = 1), Inspired Dexterous Hand, Shadow Hand, DLR-HIT II Hand, and the proposed Beyond Hand. Normalization method: Each ratio = (value of the dexterous hand)/(corresponding value of the human hand). No additional weighting was applied; all six metrics were treated equally. Note: The benchmark hands were tested under different experimental conditions reported in separate literature sources. This chart provides an indicative, qualitative comparison based on the best available data, not a strictly controlled benchmarking study.

To benchmark these metrics, all six performance indicators—DoF ratio, fingertip force ratio, weight ratio, speed ratio, compactness ratio, and frequency response ratio—were normalized to the human hand as the baseline (human hand = 1). The normalization procedure and its limitations are detailed in Section 2.3.3 [20]. The Beyond Hand was then compared against the human hand [21,22], the Inspired Dexterous Hand [23], the Shadow Hand [24], and the DLR-HIT II Hand [25]. As shown in Figure 8, the Beyond Hand achieves an overall performance profile closest to the human baseline among all compared designs. Notably, its measured peak speed exceeds the typical physiological speed limit reported for the human hand in similar tasks (speed ratio > 1) while remaining competitive in the other metrics relative to the human benchmark. In contrast, the other dexterous hands exhibit larger deviations from the human baseline and fail to match this integrated performance profile.

### 3.4. High-Dynamic Manipulation Results

#### 3.4.1. Rhythm-Game Performance

**Figure 9:**
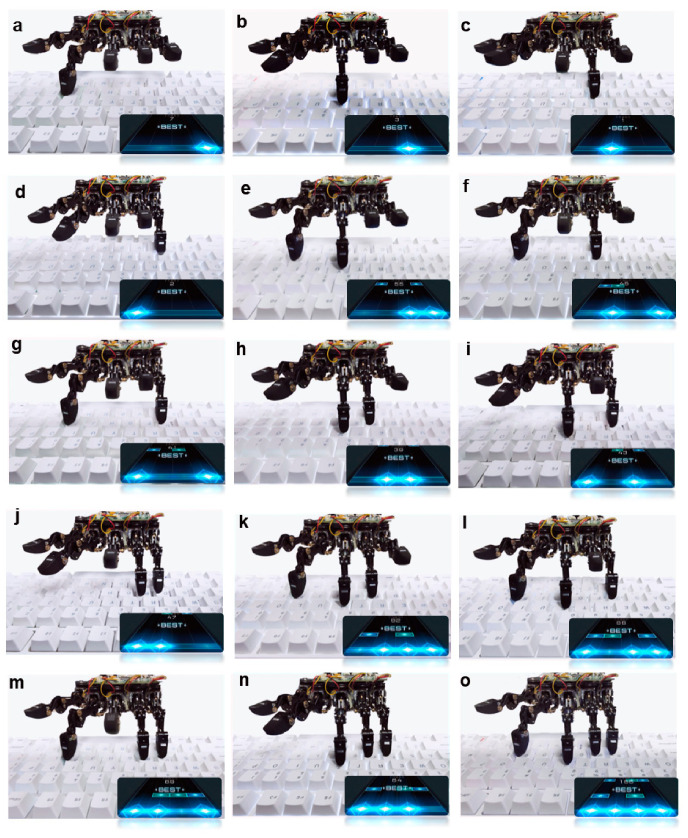
Schematic of 15 key-press patterns used in the rhythm-game task. (**a**–**d**) Single-finger presses: one finger presses one key at a time. (**e**–**j**) Two-finger presses: two fingers press two keys simultaneously. (**k**–**n**) Three-finger presses: three fingers press three keys simultaneously. (**o**) Four-finger press: four fingers press four keys simultaneously.

The 15 tapping patterns executed in this task are shown in Figure 9, spanning from single-finger presses (Figure 9a–d) through two-finger and three-finger combinations (Figure 9e–n) to four-finger simultaneous actions (Figure 9o). The robotic hand achieved an actual peak key-press density of 30 presses per second, corresponding to a maximum single-finger operating frequency of approximately 12 Hz.

**Figure 10:**
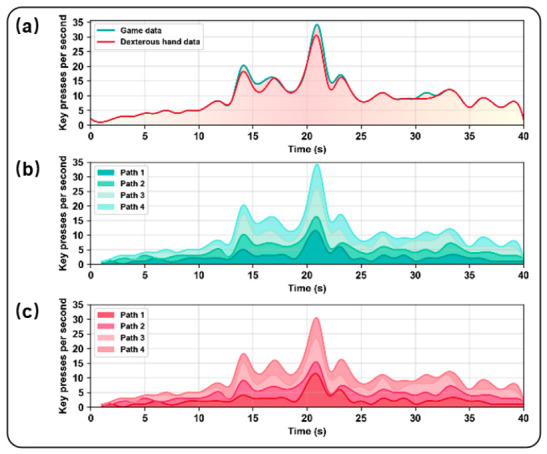
Key-press density analysis in the rhythm-game task. (**a**) Total key-press density over time: green for true slider density, red for robotic hits. (**b**) Slider density on each lane over time, with dark to light green representing lanes 1–4. (**c**) Key-press density of each finger over time, with dark to light red representing the index to little finger.

Figure 10a–c presents the key-press density analysis throughout the task. In Figure 10a, the green curve indicates the true slider density in the game, and the red curve shows the actual hit rate of the robotic hand. Figure 10b plots the time-varying slider density on the four lanes, with colors from dark to light green corresponding to lanes 1–4 respectively. Figure 10c shows the key-press density of each finger, with colors from dark to light red representing the index, middle, ring, and little fingers. The non-uniform density patterns induced by the combination of time and lane distribution clearly highlight the overall complexity of this task in terms of dynamic loading, inter-finger coordination, and timing control. The density profiles reveal highly non-uniform task demands, with instantaneous peaks exceeding 30 notes per second and frequent transitions between different finger combinations.

**Figure 11:**
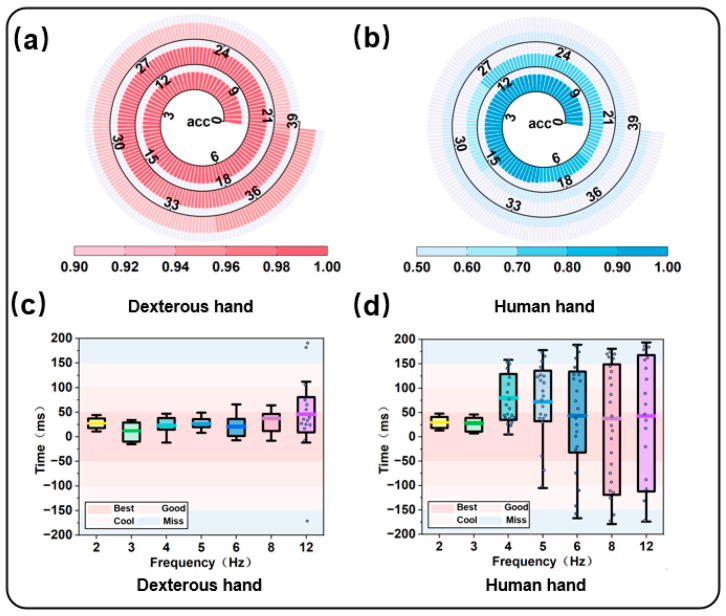
Key-press performance of the Beyond Hand and human participants in the high-dynamic rhythm-game task. (**a**) Overall key-press accuracy of the Beyond Hand. (**b**) Overall key-press accuracy of human participants. (**c**) Distribution of the Beyond Hand’s response times across different actuation frequencies. (**d**) Distribution of the human hand’s response times across different actuation frequencies.

Experimental results demonstrate that the Beyond Hand completes all required operations. Under the specific test conditions, it achieved faster task completion and more consistent control than the intermediate-level human participants in our study (*n* = 5). Throughout the task, the overall accuracy of the robotic hand remains above 90% (Figure 11a). The accuracy is maintained at 100% for operating frequencies up to 8 Hz. A slight degradation occurs at 12 Hz, though the majority of responses remain within the “Good” range of 50–100 ms. By contrast, human participants maintain high accuracy only during the initial 15 s; as the rhythm accelerates, their performance drops rapidly to around 50% toward the end of the task (Figure 11b). A video demonstration of this task is provided in the Supplementary Materials (Video S1, part 1).

The timing-distribution analysis (Figure 11c,d) provides further insight into these differences. For the robotic hand, almost all key-press responses in the 2–8 Hz range fall within the “Best” window (within ±50 ms of the target beat). At 12 Hz, only a portion shift into the “Good” range, with overall motion consistency remaining high and no obvious failure behavior. In contrast, human participants can match the rhythm reasonably well at 2–3 Hz. However, at 4–6 Hz they are already constrained by physiological speed limits, with most response times falling in the 50–150 ms range and leading to a notable drop in scores. When the frequency further increases to 6–12 Hz, their response timing becomes nearly random, and effective rhythm tracking is largely lost.

These results demonstrate that, in the structured rhythm-game task, the Beyond Hand exhibits superior temporal precision, inter-finger coordination, and high-frequency stability relative to our intermediate-level human participant cohort (*n* = 5), as evidenced by higher response accuracy, more consistent coordinated control, and more stable sustained high-frequency operation.

#### 3.4.2. Tetris-Style Manipulation Performance

The rhythm-game task demonstrated the Beyond Hand’s multi-finger coordination and sustained high-frequency capabilities. To further assess its single-finger agility and reachable workspace under spatial constraints, the Tetris-style key-press task was employed. In this task, the robotic hand keeps its wrist fixed, and the middle finger alone must perform rapid presses on three target keys located at different heights, corresponding to the “rotate” (↑), “left” (←), and “right” (→) operations. This setup challenges not only the finger’s actuation speed but also its reachable workspace and flexibility under a fixed-wrist constraint.

**Figure 12:**
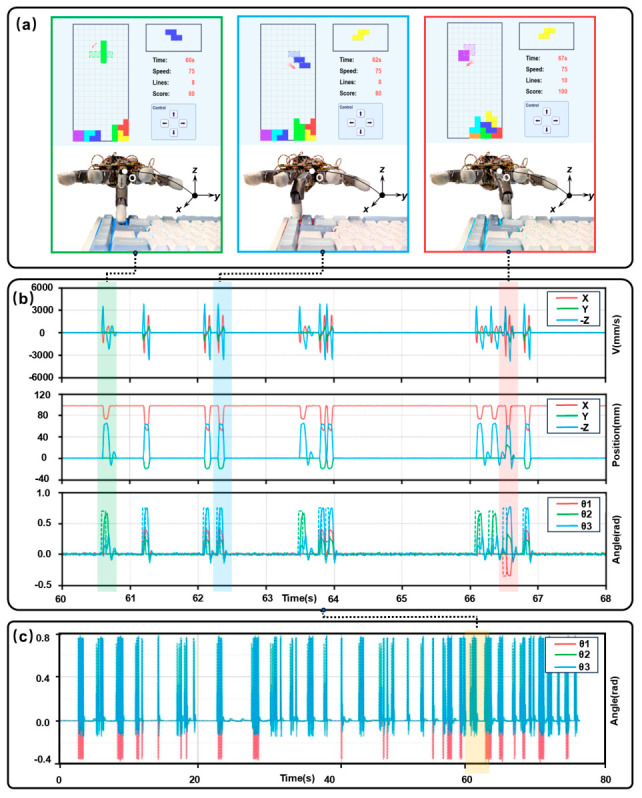
High-speed operational performance of the dexterous hand in a Tetris task. (**a**) Schematic of the task: the middle finger independently executes the three key-press actions “↑”, “←”, and “→” with the wrist kept fixed. (**b**) Fingertip angle, position, and velocity along the x-direction recorded during the 60–68 s interval. (**c**) Joint angles (θ_1_, θ_2_, θ_3_) of the middle finger recorded throughout the task.

Thanks to the systematic optimization of phalange lengths, the dexterous hand efficiently completes multi-target key-press operations without any wrist movement. As shown in Figure 12a, the middle finger accurately reaches and reliably activates all three target keys. An updated version of the hand, following further morphological optimization, successfully completes this task. A video demonstration is provided in the Supplementary Materials (Video S1, part 2).

The task imposes progressively increasing demands: blocks appear randomly and their descent speed accelerates over time (150 ms per grid cell during 0–30 s, decreasing to 75 ms during 61–75 s, as specified in Section 2.4). Figure 12b presents a detailed kinematic analysis of the middle finger during the high-intensity period from 60 to 68 s (highlighted in yellow in Figure 12c), including the time histories of the three joint angles (θ_1_, θ_2_, and θ_3_, shown in red, green, and blue curves, respectively), fingertip displacement along the x-direction, and fingertip velocity. For each of the three key-press actions marked in Figure 12a (red, green, and blue), the corresponding kinematic profiles are clearly distinguishable. The fingertip reaches a peak x-direction velocity of approximately 3000 mm/s, demonstrating excellent high-speed dynamic responsiveness. Figure 12c records the evolution of the three joint angles throughout the entire task, confirming stable and repeatable motion patterns over extended operation.

This experiment demonstrates that the dexterous hand can achieve large-range, high-speed single-finger movement and reliably execute multi-position key-press tasks, even with a fixed wrist and limited operating space. The optimized phalange ratio and transmission design provide sufficient reachability and switching speed in compact environments, enabling the middle finger to transition rapidly and continuously between different key positions.

Taken together, the results from the rhythm-game and Tetris tasks validate that the data-driven inverse design framework produces a dexterous hand capable of sustained, high-accuracy performance across a spectrum of dynamic manipulation scenarios.

## 4. Discussion

The most salient finding of this study is the convergence between design principles identified through purely data-driven analysis and those embodied in the human hand. The Cramér’s V-based association map, constructed without biological bias, independently singled out tendon-driven transmission, proximal actuator placement, and lightweight rigid structures as dominant features for high-speed dynamic performance. That the same principles characterize a system refined by millions of years of evolution strongly suggests that the high-dimensional design space of dexterous hands is governed by fundamental physical constraints admitting only a narrow set of high-performance solutions. This convergence validates the inverse design framework and offers a broader insight: well-constructed engineering datasets can recapitulate certain outcomes of evolutionary optimization, providing independent evidence for biomimetic design.

We attempted to quantitatively compare tendon routing efficiency, inertia distribution, and energy efficiency between the human hand and the Beyond Hand, but encountered substantial practical difficulties. For tendon routing efficiency, the human hand involves complex multi-articular interactions, passive elastic tissues, and nonlinear friction properties that are extremely difficult to measure or model with sufficient accuracy. For inertia distribution, the human hand’s mass properties vary significantly across individuals and cannot be directly obtained from literature without invasive measurements. For energy efficiency, there is no established standard for comparing metabolic energy consumption in biological hands with electrical power consumption in robotic hands under equivalent tasks. Therefore, in this study we focus on demonstrating morphological and kinematic convergence (e.g., degrees of freedom, finger proportions, tendon routing topology), which are more readily comparable. Quantitative comparisons of efficiency, inertia distribution, and energy consumption remain important but challenging directions for future work.

Before interpreting the association map, we note several limitations of Cramér’s V analysis. Correlation does not imply causation. Cramér’s V only quantifies the strength of a symmetric association and does not indicate any directional or causal relationship. Therefore, the inverse design framework presented here is intended as an exploratory and descriptive tool to identify potential design–performance linkages, not as a predictive or prescriptive causal model. Moreover, given the currently limited amount of available dexterous hand data, the estimated correlations may change as more data accumulate in the future. Our findings should thus be viewed as preliminary insights.

The experimental results also clarify where engineered systems can extend beyond biological capabilities. Human participants in the rhythm-game task showed a sharp performance decline above 4–6 Hz due to neuromuscular and cognitive limits. The Beyond Hand, free of these bottlenecks, sustained >90% accuracy up to 12 Hz. The uniformly stable frequency response across all 15 joints—cutoff frequencies tightly clustered around 10 Hz—further demonstrates that proximal actuation benefits the whole hand evenly. These results do not diminish the human hand’s unmatched versatility; rather, they identify timing consistency, repetition rate, and fatigue-free operation as areas where robotic systems complement biological performance.

Limitations of the game-based validation tasks. The Tetris and rhythm-game experiments primarily evaluate repetitive, high-speed tapping rather than the full scope of dexterous manipulation, such as grasp adaptation, contact-rich interaction, or handling object uncertainty. These game-based tasks were chosen intentionally to benchmark the hand’s peak speed, precision, and motion consistency under highly dynamic but structured conditions—where the contact sequence and target positions are known in advance. They are not intended to demonstrate the hand’s capability in unstructured tasks such as adaptive grasping, in-hand manipulation, or interaction with deformable or uncertain objects. Therefore, the results should be interpreted as evidence of high-speed motor performance rather than comprehensive dexterous manipulation ability. Future work will include more ecologically valid manipulation tasks (e.g., grasping unknown objects, tool use, collaborative assembly) to fully assess practical utility.

Regarding mechanical durability, after extended operation we observed some abrasion at the pin-hole interfaces of the linkages. Nevertheless, the assembly accuracy remained within acceptable limits for the intended manipulation tasks. To mitigate wear, we inserted copper sleeves at all pin-hole joints and applied sufficient lubrication, which substantially reduces friction and slows down wear. Based on these observations, we recommend routine maintenance practices, including periodic inspection of tendon tension, re-lubrication of copper sleeves, and replacement of sacrificial wear parts after extended use.

Beyond bioinspired marine robotics, origami-inspired soft robotic designs have demonstrated how simulation-driven morphological selection can achieve dynamic locomotion [26]—a design paradigm that shares conceptual parallels with the data-driven inverse design framework presented in this work.

Recent advances in underactuated tendon-driven hands with integrated tactile sensing, such as the Tactile SoftHand-A, have demonstrated that antagonistic tendon mechanisms combined with 3D-printed tactile structures can achieve robust grasping across diverse objects [27]. This work provides a relevant reference for future iterations of the Beyond Hand, suggesting that tactile embodiment can be incorporated without sacrificing mechanical simplicity.

Several specific limitations point to future directions. First, the Beyond Hand lacks tactile sensing, which prevents fine force modulation and adaptive grasping [28,29]. Second, the association map, while effective, depends on the current dataset’s composition and size; its statistical resolution will improve as more dexterous hand designs are added. Third, the validation tasks, though demanding, remain structured; unstructured environments with varied objects and physical interactions need to be addressed. Integrating tactile sensing and real-time feedback control into the architecture will be a necessary next step. Finally, the convergence observed here suggests that inverse design frameworks, applied to larger and more diverse datasets, could eventually generate quantitative hypotheses in comparative biomechanics, closing the loop between engineering and biology.

Limitations regarding computational cost, generalizability, and multi-objective optimization. The proposed inverse design framework is computationally lightweight: the core Cramér’s V analysis on the dataset of 77 dexterous hands completes within seconds on a standard PC (e.g., Intel i5, 16 GB RAM) using Python (3.11.7)’s SciPy library. The dominant effort is the one-time dataset construction (literature search, data extraction, normalization), which is performed offline. The framework can be extended to other robotic platforms (e.g., humanoid or quadruped robots), as the principle of correlating categorical design variables with performance metrics is platform-independent. However, the framework does not inherently resolve trade-offs when performance metrics conflict (e.g., speed vs. force, weight vs. durability). It identifies which design variables influence each metric, providing a basis for informed trade-off decisions. When objectives are non-conflicting, simultaneous optimization is straightforward; when conflicts exist, explicit multi-objective optimization (e.g., Pareto front analysis) would be required. The latter is not implemented in the current work and remains an important future direction.

## 5. Conclusions

This study addressed the challenge of simultaneously achieving human-like scale and dexterity with elevated dynamic performance in dexterous hand design. By constructing a quantitative association map between design variables and performance metrics, we established a data-driven inverse design framework that translates explicit dynamic targets into an optimized hardware configuration—replacing the empirical trial-and-error paradigm. The resulting Beyond Hand preserves a 20-DoF anthropomorphic form while embodying the biomechanical principles of tendon-driven transmission, proximal actuation, and lightweight rigid structures.

Standardized frequency-response tests across all 15 finger joints revealed stable and well-balanced dynamic characteristics, with magnitude attenuation below 3 dB at 14 Hz and cutoff frequencies tightly clustered around 10 Hz. In high-dynamic manipulation tasks, the Beyond Hand maintained over 90% overall accuracy in rhythm-game operations at frequencies up to 12 Hz, achieved better performance than the human participants in our study under the same structured task conditions The Tetris-style single-finger task further demonstrated rapid, reliable multi-target key-press capability, with fingertip velocities reaching approximately 3000 mm/s under progressively tightening time constraints and a fixed wrist.

Beyond validating specific performance targets, this work shows that purely data-driven analysis can independently recover design principles convergent with those of biological evolution, underscoring the value of quantitative performance-driven methodologies. The proposed framework is scalable and will be refined as the underlying design dataset grows. Future extensions will incorporate tactile sensing, adaptive grasping in unstructured environments, and real-time feedback control [30]. The Beyond Hand thus serves as both a validation platform and a blueprint for next-generation high-performance biomimetic robotic hands, with promising applications in service robotics, rehabilitation, high-speed industrial manipulation, and human–robot collaborative scenarios.

We also acknowledge that the human comparison in this study involved only five intermediate-level participants performing a structured rhythm-game task. Therefore, the finding that the Beyond Hand outperformed these participants should not be overgeneralized to all human capabilities. The human hand remains unmatched in versatility, sensory feedback, and adaptive manipulation.

## Figures and Tables

**Table 1:** Mapping between biological hand features, biomimetic principles, and engineering design choices in the Beyond Hand.

Biological Hand Feature	Biomimetic Principle	Engineering Design Choice
Flexor/extensor tendons	Proximal actuation for low distal inertia; tendon compliance for energy-efficient force transmission	First-level transmission: Tendon-driven mode
Multi-articular tendon routing across PIP/DIP/MCP joints	Natural mechanical coupling across joints	Underactuated design with coupled joints via tendon routing
Rigid phalangeal bones articulating at synovial joints	Rigid segments with low-friction articulation	Second-level transmission: Linkage-driven mode
Cortical and trabecular bone tissue	Lightweight, high-strength structural framework	Structural material: Aluminum alloy
Muscle bellies located in forearm, acting on hand via long tendons	Mass concentrated proximally to reduce distal inertia and improve dynamic response	Actuator placement: All actuators in forearm; total 20-DoF configuration

## Data Availability

The original contributions presented in this study are included in the article and its Supplementary Materials. Further inquiries can be directed to the corresponding authors.

## Supplementary Materials

The following supporting information can be downloaded at: https://www.mdpi.com/article/10.3390/biomimetics11060434/s1, Video S1: part 1: Rhythm-game task demonstration; part 2: Tetris-task demonstration; part 3: Complete dataset of dexterous hand designs.
